# Sensory Loss among Cognitively Impaired Spousal Caregivers: Findings from the Health and Retirement Study

**DOI:** 10.1093/geroni/igaf122.3324

**Published:** 2025-12-31

**Authors:** Shaoqing Ge, Qingyi Jasmine Zeng

**Affiliations:** University of Texas at Austin School of Nursing, Austin, Texas, United States; The University of Texas School of Nursing, Austin, Texas, United States

## Abstract

With the global trend of aging, more older adults are assuming caregiving roles for their loved ones. Sensory loss and cognitive decline are prevalent among older adult caregivers, exacerbating the stress and burden of caregiving and leading to increased physical and emotional strain. Previous studies lacked an understanding of the inter-relationship between sensory loss, cognitive impairment, and caregiving. This study addresses this gap by analyzing data from the 2020 Health and Retirement Study (HRS), focusing on cognitively impaired spousal caregivers with self-reported sensory data. Using the Langa-Weir (LW) algorithm, among 926 spousal caregivers, 157 (17%) were categorized as cognitively impaired but not dementia (CIND), and 29 (3%) had dementia. Among cognitively impaired spousal caregivers, 116 rated hearing function as “good and above,” while 70 rated it as “fair/poor.” Additionally, 89 rated their eyesight as “good and above,” and 95 rated it as “fair/poor.” Although ANOVA analysis showed a significant relationship between race and hearing status, being African American or Hispanic/Latino did not significantly increase the odds of having fair or poor hearing in the logistic regression models. Conversely, the odds of having “good and above” eyesight decreased among African American (OR = 0.23, 95% CI = 0.07–0.73, p<.05) and Hispanic caregivers (OR = 0.09, 95% CI = 0.02–0.39, p<.01). Other factors, including age, gender, education, income, working status, and Medicaid status, were non-significant in affecting the odds of better hearing or vision. These findings underscore the need for a comprehensive understanding of the risk factors contributing to sensory loss and associated challenges among cognitively impaired spousal caregivers.

